# Ion Slip Effect on Viscoelastic Fluid Flow past an Impulsively Started Infinite Vertical Plate Embedded in a Porous Medium with Chemical Reaction

**DOI:** 10.1155/2014/481308

**Published:** 2014-09-11

**Authors:** Rita Choudhury, Paban Dhar

**Affiliations:** Department of Mathematics, Gauhati University, Guwahati, Assam, India

## Abstract

This paper presents the study of convective heat and mass transfer characteristics of an incompressible MHD viscoelastic fluid past an infinite vertical plate immersed in a porous medium with chemical reaction and ion slip effects. Highly nondimensional governing equations are solved analytically by perturbation scheme. The analytical expressions for velocity, shearing stress, temperature, concentration, rate of heat transfer, and mass transfer are obtained. Also, graphical representations have been carried out for velocity field and shearing stress to investigate the effects of viscoelasticity and the effects of ion slip on the fluid flow in combination with other physical parameters involved in the solution.

## 1. Introduction

Analysis of flow over a vertical surface with simultaneous heat and mass transfer from different geometrics embedded in porous media has many engineering and geophysical applications such as geothermal reservoirs, drying of porous solids, thermal insulation, enhanced oil recovery, packed-bed catalytic reactors, and underground energy transport. The various aspects of the pioneering work of Sakiadis [1] on continuous surfaces have been investigated by many authors. Again, the studies of magnetohydrodynamics (MHD) flow for electrically conducting fluid past a porous vertical surface are important from technological point of view. Pioneering work of Hartmann [2] on the flow fluid under the influence of uniform transverse magnetic field has led to an extensive study on MHD flow problems by several researchers. These studies have a bearing on industrial applications such as MHD power generators, MHD pumps, accelerators, aerodynamic heating, electrostatic precipitation, polymer technology, petroleum industry, purification of crude oil and fluid droplets and sprays. Furthermore, a lot of research work concerning the MHD flow has been obtained under different physical effects by the investigators like Nigam and singh [3], Makinde [4], Muthucumaraswamy et al. [5], Geindreau and Auriault [6] and Apelblat [7]. In most cases, the Hall and ion slip terms were ignored in applying Ohm's law, as they have no marked effect for small and moderate values of the magnetic field. However, the current trend for the application of magnetohydrodynamics is towards a strong magnetic field, so that the influence of electromagnetic force is noticeable as studied by Cramer and Pai [8]. Under these conditions, the Hall current and ion slip are important and they have a marked effect on the magnitude and direction of the current density and consequently on the magnetic force term. The effects of Hall current on MHD free convection flow along a vertical surface with or without mass transfer have been studied by number of authors: Sato [9], Tani [10], Pop [11], Raptis and Ram [12], Hossain et al. [13–15], Pop and Watanabe [16], Acharya et al. [17], Aboeldahab and Elbarbary [18], Acharya et al. [19], and so forth. Hall current with ion slip has important engineering applications in the problem of magnetohydrodynamics generators and Hall accelerators as well as in flight magnetoaerodynamics. Attia [20] has studied the unsteady Couette flow with heat transfer considering ion slip. Furthermore, Attia [21] has investigated the effects of heat and mass transfer in elasticoviscous fluid past an impulsively started infinite vertical plate by considering ion slip.

In the present study, we propose to investigate the effects of simultaneous heat and mass transfer on the free convection MHD flow of an incompressible electrically conducting viscoelastic fluid characterized by Walters liquid (Model B′) past an impulsively started infinite vertical plate embedded in a porous medium considering Hall and ion slip effect into account with chemical reaction. Solutions are presented in graphical forms for various parametric values entering into the fluid flow region.

The constitutive equation for Walters liquid (Model B′) is

(1) σik=−pgik+σik′,σ′ik=2η0eik−2k0e′ik,

where *σ*_*ik*_ is the stress tensor, *p* is isotropic pressure, *g*_*ik*_ is the metric tensor of a fixed coordinate system *x* ^*i*^,  *v*_*i*_ is the velocity vector, and the contravariant form of *e* ^′*ik*^ is given by

(2) $$\begin{array}{c} e^{\prime ik}=\frac{\partial e^{ik}}{\partial t}+v^{m}e_{,m}^{ik}-v_{,m}^{k}e^{im}-v_{,m}^{i}e^{mk}. \end{array}$$

It is the convected derivative of the deformation rate tensor *e* ^*ik*^ defined by

(3) $$\begin{array}{c} 2e^{ik}=v_{i,k}+v_{k,i}. \end{array}$$

Here, *η*_0_ is the limiting viscosity at the small rate of shear which is given by

(4) $$\begin{array}{c} \eta_{0}=\overset{\infty }{\underset{0}{\int }}N\left(\tau \right)d\tau ,\;\;k_{0}=\overset{\infty }{\underset{0}{\int }}\tau N\left(\tau \right)d\tau \end{array}$$

with *N*(*τ*) being the relaxation spectrum. This idealized model is a valid approximation of Walters liquid (Model B′) taking very short memories into account so that terms involving

(5) $$\begin{array}{c} \overset{\infty }{\underset{0}{\int }}t^{n}N\left(\tau \right)d\tau ,\;n\geq 2, \end{array}$$

have been neglected.

## 2. Mathematical Formulation

**Figure 1:**
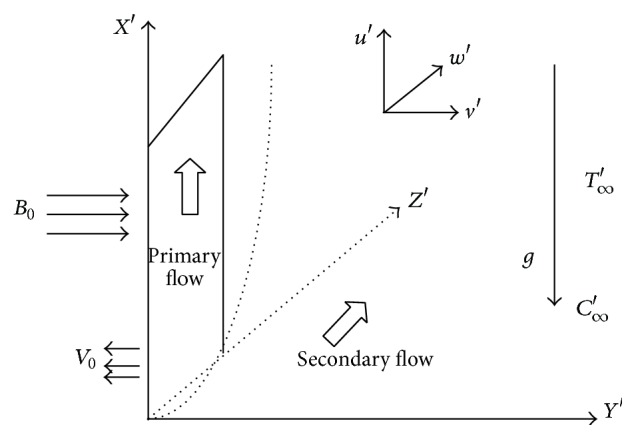
Physical configuration of the problem.

We consider the unsteady flow of an incompressible electrically conducting viscoelastic fluid past an impulsively started infinite vertical porous plate with oscillating temperature and concentration embedded in a porous medium in the presence of a transversely imposed magnetic field with a coordinate system (*x*′, *y*′, *z*′), where *x*′-axis is oriented vertically upwards along the plate and *y*′-axis is taken as normal to the plane of the plate while *z*′-axis is taken along the width of the plate as shown in Figure 1. The plate starts moving in its own plane with velocity *U*_0_. Since the plate is of infinite length, all the physical variables are function of *y*′ and *t*′ only. In order to make the flow model more ideal, the investigation is restricted to the following assumptions.

(i) The plate is electrically nonconductive and is subjected to a constant suction velocity *V*_0_.
(ii) The magnetic Reynolds number is so small that the induced magnetic field can be neglected. Also, the electrical conductivity of the fluid is reasonably low and hence the Ohmic dissipation may be neglected.
(iii) The electron pressure is constant.
(iv) The energy dissipated due to internal friction of the fluid particles is negligible.
(v) In the absence of an externally applied electric field and with negligible effects of polarization of the ionized gas, the electric field vector is assumed to be zero.

With the foregoing assumptions and under the usual boundary layer and Boussinesq approximations, the governing equations of motion are as follows.

Continuity equation is

(6) $$\begin{array}{c} \frac{\partial v^{\prime }}{\partial y^{\prime }}=0⟹v^{\prime }=-V_{0}. \end{array}$$

Momentum equation is

(7)   ∂u′∂t′+v′∂u′∂y′=η0ρ∂2u′∂y′2−k0ρ(∂3u′∂t′∂y′2+v′∂3u′∂y′3) +gβ(T′−T∞′)+gβ′(C′−C∞′) −σB02[(1+BeBi)u′+Bew′]ρ[(1+BeBi)2+Be2]−υu′K′,∂w′∂t′+v′∂w′∂y′=η0ρ∂2w′∂y′2−k0ρ(∂3w′∂t′∂y′2+v′∂3w′∂y′3) −σB02[(1+BeBi)w′−Beu′]ρ[(1+BeBi)2+Be2]−υw′K′.

Energy equation is

(8) $$\begin{array}{c} \frac{\partial T^{\prime }}{\partial t^{\prime }}+v^{\prime }\frac{\partial T^{\prime }}{\partial y^{\prime }}=\frac{K_{T}}{\rho C_{p}}\frac{\partial^{2}T^{\prime }}{\partial y^{\prime 2}}. \end{array}$$

Concentration equation is

(9) $$\begin{array}{c} \frac{\partial C^{\prime }}{\partial t^{\prime }}+v^{\prime }\frac{{\partial C}^{\prime }}{\partial y^{\prime }}=D\frac{{\partial^{2}C}^{\prime }}{{\partial y}^{\prime 2}}-K_{l}\left(C^{\prime }-C_{\infty }^{\prime }\right), \end{array}$$

where

(10) $$\begin{array}{c} T^{\prime }-T_{\infty }^{\prime }=\theta^{\prime },\;\;C^{\prime }-C_{\infty }^{\prime }=\phi^{\prime } \end{array}$$

subject to the boundary conditions which are

(11) $$\begin{array}{c} y^{\prime }=0\text{:}\;u^{\prime }=U_{0},\;\;w^{\prime }=0,\\ \theta^{\prime }=ae^{i\omega^{\prime }t^{\prime }},\;\;\phi^{\prime }=be^{i\omega^{\prime }t^{\prime }},\\ y^{\prime }⟶\infty \text{:}\;u^{\prime }⟶0,\;\;w^{\prime }⟶0,\\ \theta^{\prime }⟶0,\;\;\phi^{\prime }⟶0. \end{array}$$

For the sake of normalization of the flow model, we introduce the following nondimensional quantities:

(12) η=V0νy′,  t=V02t′4ν,  u=u′V0,w=w′V0,  θ=θ′a,  ϕ=ϕ′b,  Pr=ρνCpKT,Gr=gβν(T′−T∞′)aV03,  M=σνB02ρV02,  K=K′V02ν2,Sc=νD,  γ=νKlV02k=k0V02ρν2,  Gm=gβ′ν(C′−C∞′)bV03.

The physical parameters shown are defined in the nomenclature.

We get the following governing equations which are dimensionless:

(13) 14∂u∂t−∂u∂η=∂2u∂η2−k[14∂3u∂t∂η2−∂3u∂η3] −M{(1+BeBi)u+Bew}(1+BeBi)2+Be2 +Grθ+Gmϕ−uK,

(14) 14∂w∂t−∂w∂η=∂2w∂η2−k[14∂3w∂t∂η2−∂3w∂η3]  −M[(1+BeBi)w−Beu](1+BeBi)2+Be2−wK,

(15) 14∂θ∂t−∂θ∂η=1Pr∂2θ∂η2

(16) 14∂ϕ∂t−∂ϕ∂η=1Sc∂2ϕ∂η2−γϕ.

The relevant boundary conditions are

(17) $$\begin{array}{c} \eta =0\text{:}\;u=1,\;\;w=0,\\ \theta =e^{i\omega t},\;\;\phi =e^{i\omega t},\\ \eta ⟶\infty \text{:}\;u⟶0,\;\;w⟶0,\\ \theta ⟶0,\;\;\phi ⟶0. \end{array}$$

### 2.1. Method of Solution

Introducing the complex variable

(18) $$\begin{array}{c} \psi =u\left(\eta ,t\right)+iw\left(\eta ,t\right), \end{array}$$

where$\;i=\sqrt{-1}$, (13) and (14) transform to single partial differential equation

(19) 14∂ψ∂t−∂ψ∂η=∂2ψ∂η2−k[14∂3ψ∂t∂η2−∂3ψ∂η3] −M[(1+BeBi)−iBe]ψ(1+BeBi)2+Be2−ψK+Grθ+Gmϕ

subject to boundary conditions

(20) $$\begin{array}{c} \eta =0\text{:}\;\psi =1,\;\;\theta =e^{i\omega t},\;\;\phi =e^{i\omega t},\\ \eta ⟶\infty \text{:}\;\psi ⟶0,\;\;\theta ⟶0,\;\;\phi ⟶0. \end{array}$$

In order to solve (15), (16), and (19) under the boundary conditions of (20), we assume that

(21) $$\begin{array}{c} \theta \left(\eta ,t\right)=\theta_{0}\left(\eta \right)e^{i\omega t}, \end{array}$$

(22) $$\begin{array}{c} \phi \left(\eta ,t\right)=\phi_{0}\left(\eta \right)e^{i\omega t}, \end{array}$$

(23) $$\begin{array}{c} \psi \left(\eta ,t\right)=\psi_{0}\left(\eta \right)e^{i\omega t}. \end{array}$$

Using (21) and (22) into (15) and (16), we get

(24) $$\begin{array}{c} \theta_{0}=e^{-(\eta /2)(\text{Pr}+\sqrt{{\text{Pr}}^{2}+i\omega \text{Pr}})},\\ \phi_{0}=e^{-(\eta /2)(\text{Sc}+\sqrt{{\text{Sc}}^{2}+(i\omega \text{Sc}+4\gamma \text{Sc})})}. \end{array}$$

Using *θ*_0_, *ϕ*_0_ in (21) and (22), respectively, we get the solutions for the temperature and the concentration fields which are as follows:

(25) $$\begin{array}{c} \theta =e^{i\omega t-(\eta /2)(\text{Pr}+\sqrt{{\text{Pr}}^{2}+i\omega \text{Pr}})}\\ \phi =e^{i\omega t-(\eta /2)(\text{Sc}+\sqrt{{\text{Sc}}^{2}+(i\omega \text{Sc}+4\gamma \text{Sc})})}. \end{array}$$

Substituting the values of (23) in (19), we get

(26) kψ0′′′+(1−ikω4)ψ0′′+ψ0′−(a1−ia2)ψ0  =−Grθ0−Gmϕ0,

where primes denote differentiation with respect to *η*.

The corresponding transformed boundary conditions are

(27) $$\begin{array}{c} \eta =0\text{:}\;\psi_{0}=e^{-i\omega t},\\ \eta ⟶\infty \text{:}\;\psi_{0}⟶0. \end{array}$$

To solve (26), we use the multiparameter perturbation technique as follows:

(28) $$\begin{array}{c} \psi_{0}=\psi_{00}\left(\eta \right)+k\psi_{01}\left(\eta \right), \end{array}$$

as *k* ≪ 1, for small shear rate [22].

Using (28) in (26) and equating the coefficients of like powers of *k*, we get

(29) $$\begin{array}{c} \psi_{00}^{\prime \prime }+\psi_{00}^{\prime }-\left(a_{1}-ia_{2}\right)\psi_{00}=-\text{Gr}\theta_{0}-\text{Gm}\phi_{0},\\ \psi_{01}^{\prime \prime }+\psi_{01}^{\prime }-\left(a_{1}-ia_{2}\right)\psi_{01}=\frac{i\omega }{4}\psi_{00}^{\prime \prime }-\psi_{00}^{\prime \prime \prime }, \end{array}$$

subject to modified boundary condition

(30) $$\begin{array}{c} \eta =0,\;\;\psi_{00}=e^{-i\omega t},\;\;\psi_{01}=0,\\ \eta ⟶\infty ,\;\;\psi_{00}⟶0,\;\;\psi_{01}⟶0. \end{array}$$

Solving (29) under the boundary conditions (30), we get

(31) ψ0=(c1e−α6η+c3e−α2η+c5e−α4η) +i(c2e−α6η+c4e−α2η+c6e−α4η).

From (23), we get

(32) ψ=ψ0eiωt={(c1e−α6η+c3e−α2η+c5e−α4η)hhl+i(c2e−α6η+c4e−α2η+c6e−α4η)}eiωt.

Now, equating the real and imaginary part, we get the axial and transverse components of velocity as follows:

(33) u=(c1e−α6η+c3e−α2η+c5e−α4η)cos⁡⁡ωt −(c2e−α6η+c4e−α2η+c6e−α4η)sin⁡ωt,w=(c2e−α6η+c4e−α2η+c6e−α4η)cos⁡⁡ωt +(c1e−α6η+c3e−α2η+c5e−α4η)sin⁡ωt.

The constants are obtained but are not given here for the sake of brevity.

#### 2.1.1. Skin Friction

The axial component of the shearing stress at the plate for primary velocity is

(34) σ1=(∂u∂η−k[14∂2u∂t∂η−∂2u∂η2])η=0=−(c1α6+c3α2+c5α4)cos⁡⁡ωt +(c2α6+c4α2+c6α4)sin⁡ωt −k[14{(c1α6+c3α2+c5α4)ωsin⁡ωthhhhhhhlh+(c2α6+c4α2+c6α4)ωcos⁡⁡ωt}hhhhhh−{(c1α62+c3α22+c5α42)cos⁡⁡ωthhhhhhhhh−(c2α62+c4α22+c6α42)sin⁡ωt}14].

The transverse component of the shearing stress at the plate for secondary velocity is

(35) σ2=(∂w∂η−k[14∂2w∂t∂η−∂2w∂η2])η=0=−(c2α6+c4α2+c6α4)cos⁡⁡ωt −(c1α6+c3α2+c5α4)sin⁡ωt −k[14{(c2α6+c4α2+c6α4)ωsin⁡ωthhhhhhhh−(c1α6+c3α2+c5α4)ωcos⁡⁡ωt}hhhlhh−(c2α62+c4α22+c6α42)cos⁡⁡ωthhlhhh+14(c1α62+c3α22+c5α42)sin⁡ωt].

The rate of heat transfer in terms of the Nusselt number is given by

(36) $$\begin{array}{c} \text{Nu}=-{\left(\frac{\partial \theta }{\partial \eta }\right)}_{\eta =0}=\frac{1}{2}\left(\text{Pr}+\sqrt{{\text{Pr}}^{2}+i\omega \text{Pr}}\right)e^{i\omega t}. \end{array}$$

The rate of mass transfer coefficient in terms of the Sherwood number is given by

(37) $$\begin{array}{c} \text{Sh}={\left(\frac{\partial \phi }{\partial \eta }\right)}_{\eta =0}=-\frac{1}{2}\left(\text{Sc}+\sqrt{{\text{Sc}}^{2}+\left(i\omega \text{Sc}+4\gamma \text{Sc}\right)}\right)e^{i\omega t}. \end{array}$$

## 3. Result and Discussion

**Figure 2:**
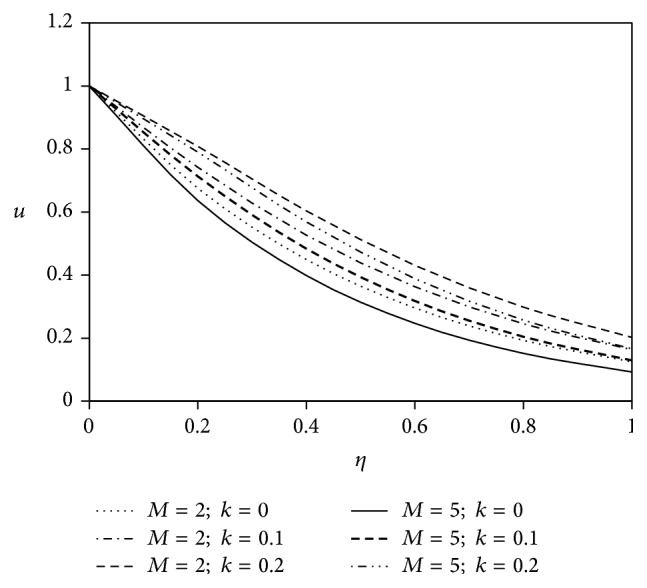
Variation of primary velocity *u* against *η* for Pr = 3, Gr = 5, Gm = 5, Sc = 5, *γ* = 0.5, Be = 1, and Bi = 1.

**Figure 3 fig3:**
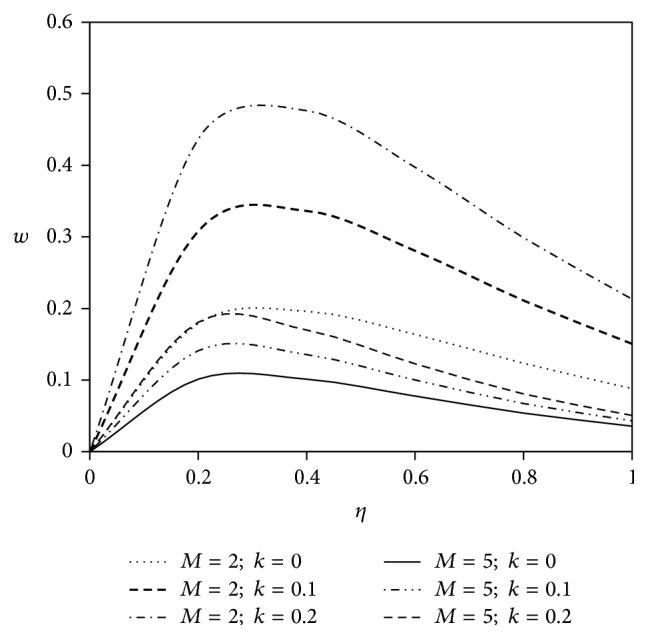
Variation of secondary velocity *w* against *η* for Pr = 3, Gr = 5, Gm = 5, Sc = 5, *γ* = 0.5, Be = 1, and Bi = 1.

**Figure 4 fig4:**
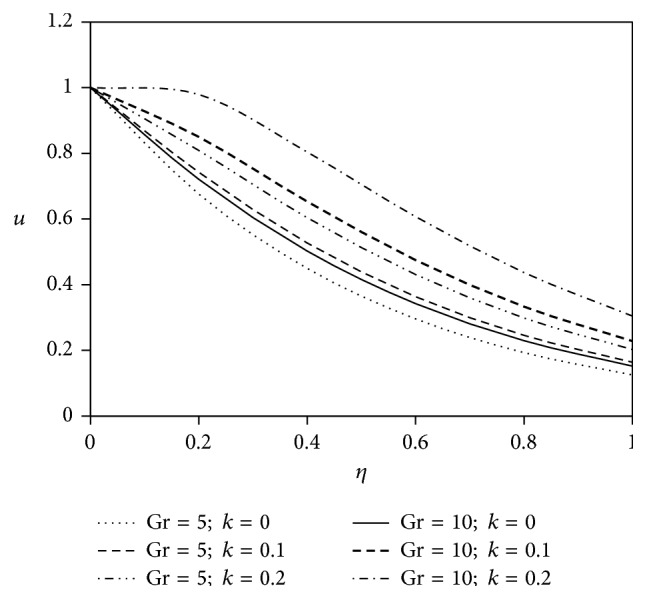
Variation of primary velocity *u* against *η* for Pr = 3, *M* = 2, Gm = 5, Sc = 5, *γ* = 0.5, Be = 1, and Bi = 1.

**Figure 5 fig5:**
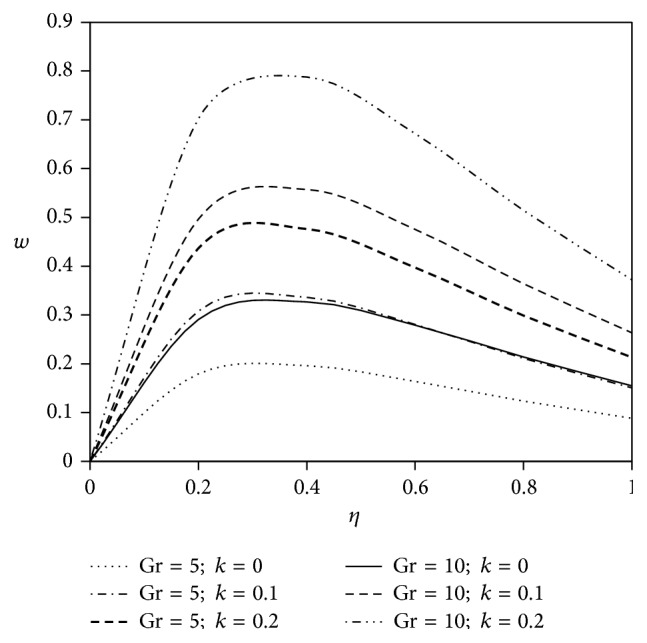
Variation of secondary velocity *w* against *η* for Pr = 3, *M* = 2, Gm = 5, Sc = 5, *γ* = 0.5, Be = 1, and Bi = 1.

**Figure 6 fig6:**
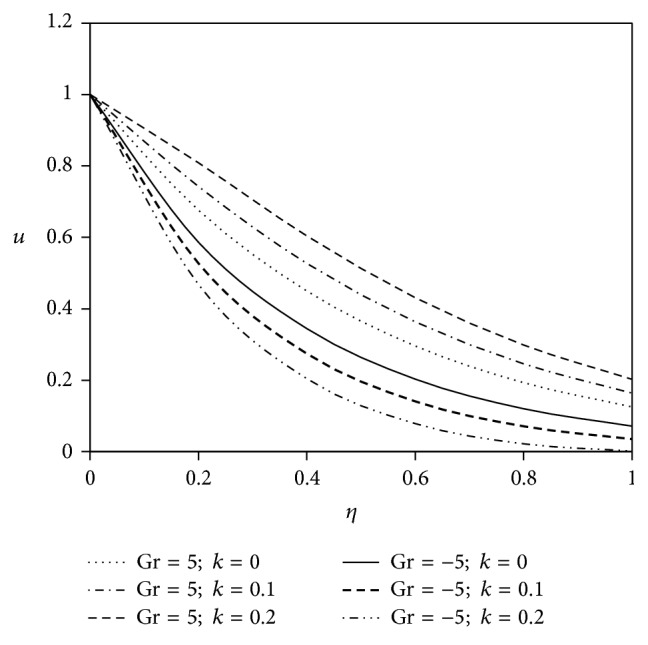
Variation of primary velocity *u* against *η* for Pr = 3, *M* = 2, Gm = 5, Sc = 5, *γ* = 0.5, Be = 1, and Bi = 1.

**Figure 7 fig7:**
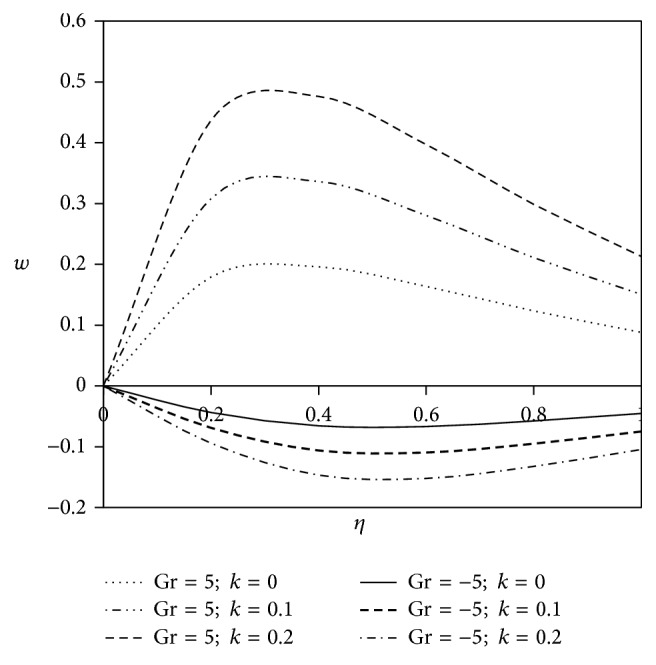
Variation of secondary velocity *w* against *η* for Pr = 3, *M* = 2, Gm = 5, Sc = 5, *γ* = 0.5, Be = 1, and Bi = 1.

**Figure 8 fig8:**
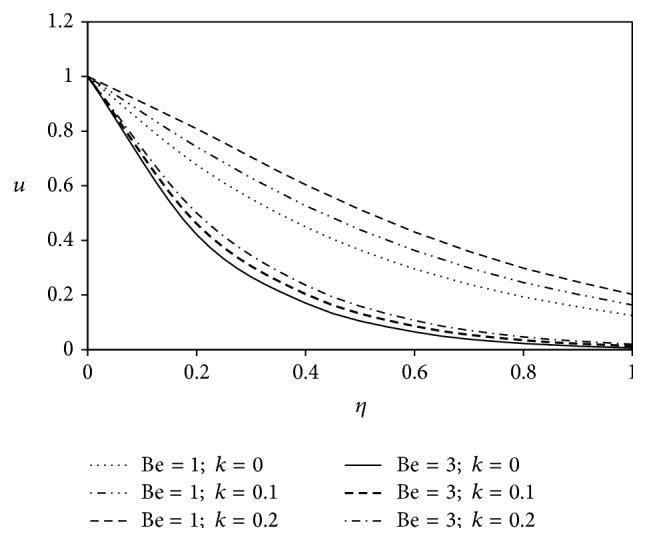
Variation of primary velocity *u* against *η* for Pr = 3, *M* = 2, Gr = 5, Gm = 5, Sc = 5, *γ* = 0.5, and Bi = 1.

**Figure 9 fig9:**
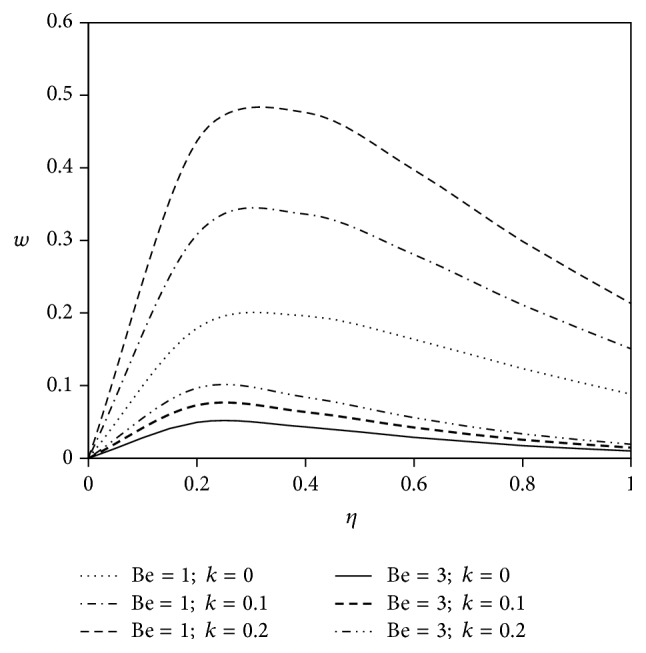
Variation of secondary velocity *w* against *η* for Pr = 3, *M* = 2, Gr = 5, Gm = 5, Sc = 5, *γ* = 0.5, and Bi = 1.

**Figure 10 fig10:**
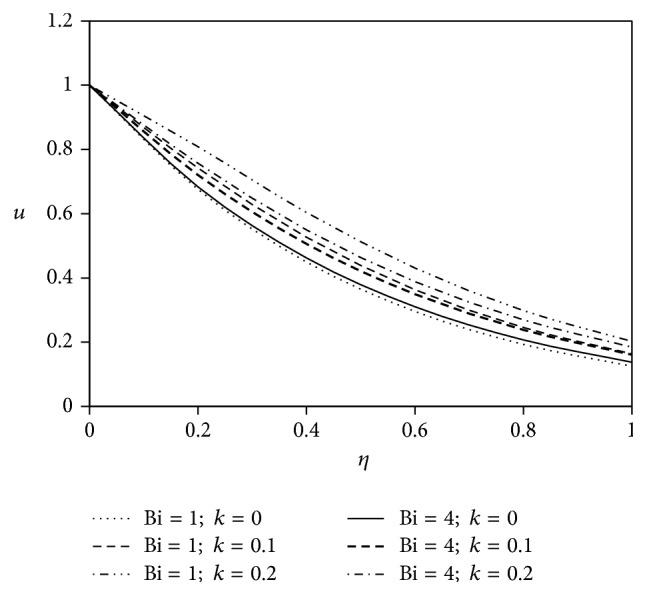
Variation of primary velocity *u* against *η* for Pr = 3, *M* = 2, Gr = 5, Gm = 5, Sc = 5, *γ* = 0.5, and Be = 1.

**Figure 11:**
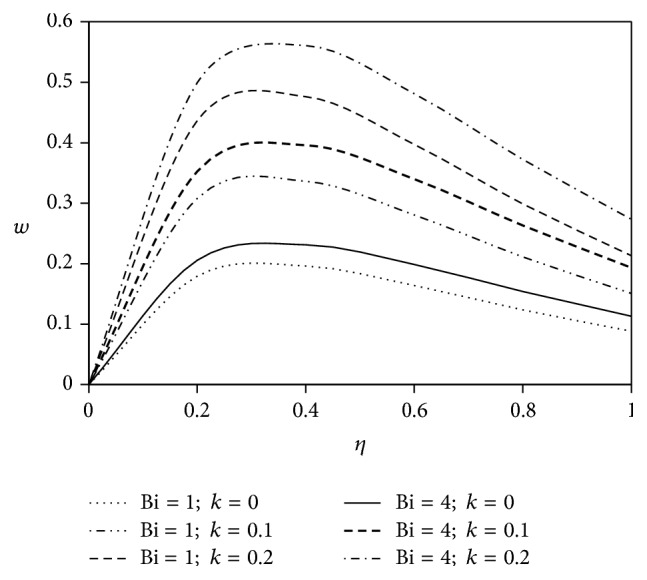
Variation of secondary velocity *w* against *η* for Pr = 3, *M* = 2, Gr = 5, Gm = 5, Sc = 5, *γ* = 0.5, and Be = 1.

**Figure 12:**
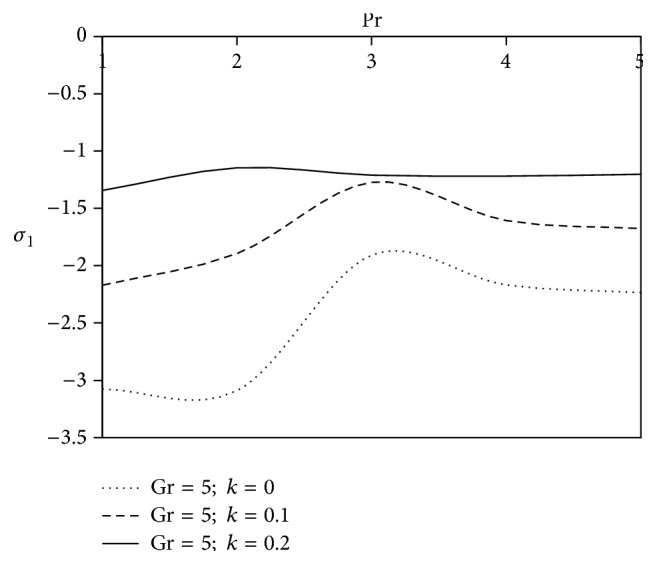
Variation of axial shearing stress *σ*_1_ against Pr for *M* = 2, Gm = 5, Sc = 5, *γ* = 0.5, Be = 1, and Bi = 1.

**Figure 13 fig13:**
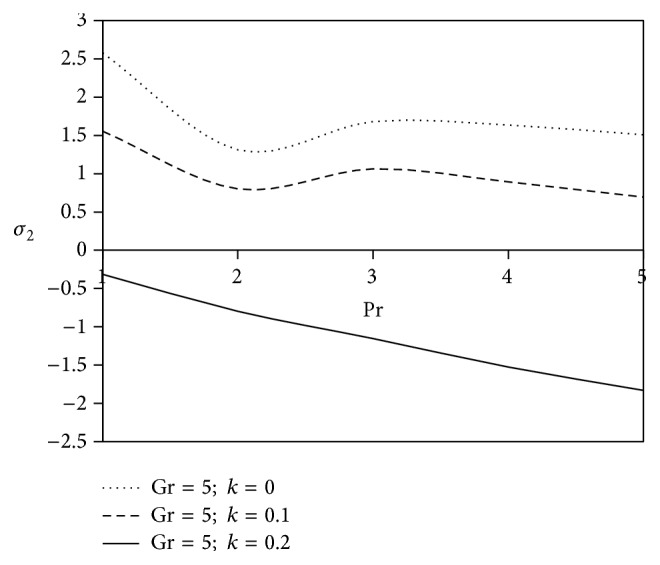
Variation of transverse shearing stress *σ*_2_ against Pr for *M* = 2, Gm = 5, Sc = 5, *γ* = 0.5, Be = 1, and Bi = 1.

**Figure 14 fig14:**
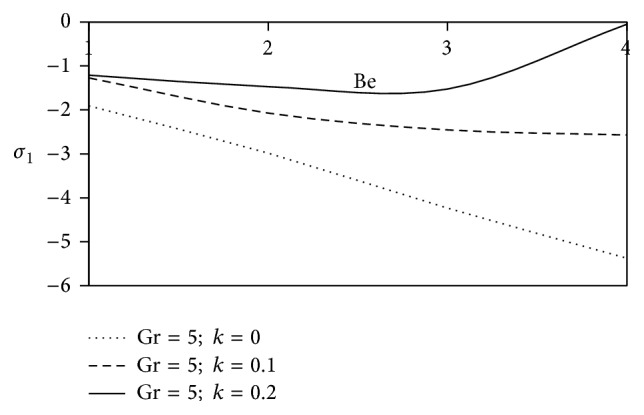
Variation of axial shearing stress *σ*_1_ against Be for Pr = 3, *M* = 2, Gm = 5, Sc = 5, *γ* = 0.5, and Bi = 1.

**Figure 15 fig15:**
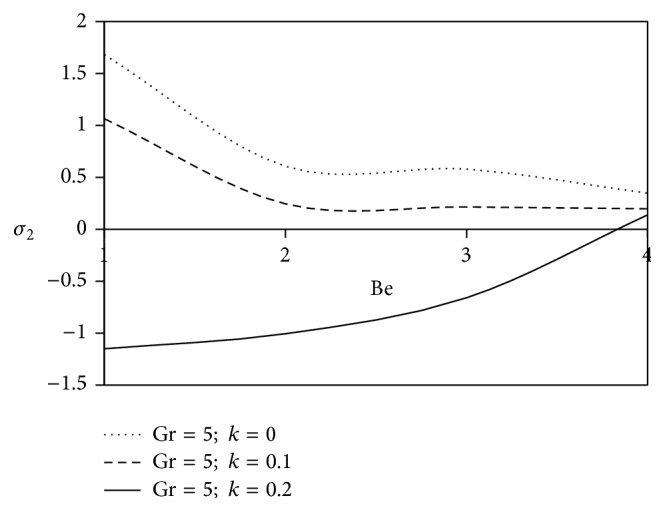
Variation of transverse shearing stress *σ*_2_ against Be for Pr = 3, *M* = 2, Gm = 5, Sc = 5, *γ* = 0.5, and Bi = 1.

**Figure 16 fig16:**
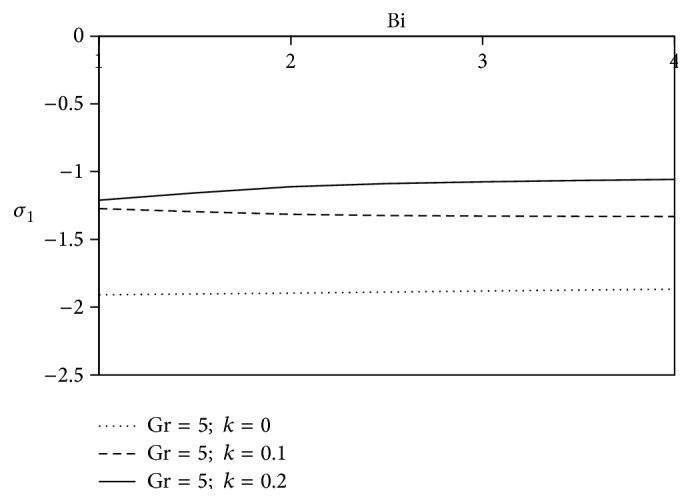
Variation of axial skin friction *σ*_1_ against Bi for Pr = 3, *M* = 2, Gm = 5, Sc = 5, *γ* = 0.5, and Be = 1.

**Figure 17 fig17:**
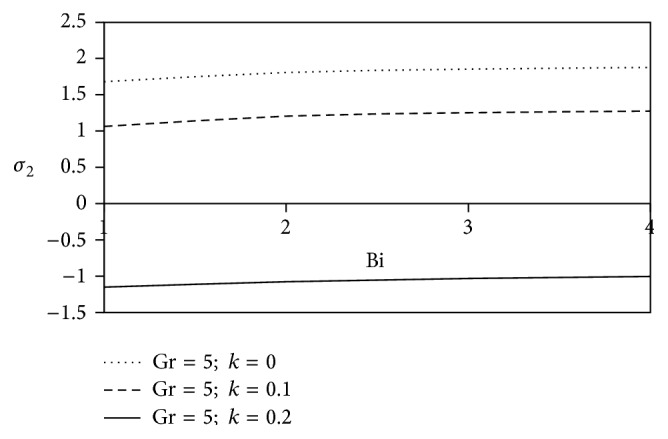
Variation of transverse skin friction *σ*_2_ against Bi for Pr = 3, *M* = 2, Gm = 5, Sc = 5, *γ* = 0.5, and Be = 1.

**Figure 18 fig18:**
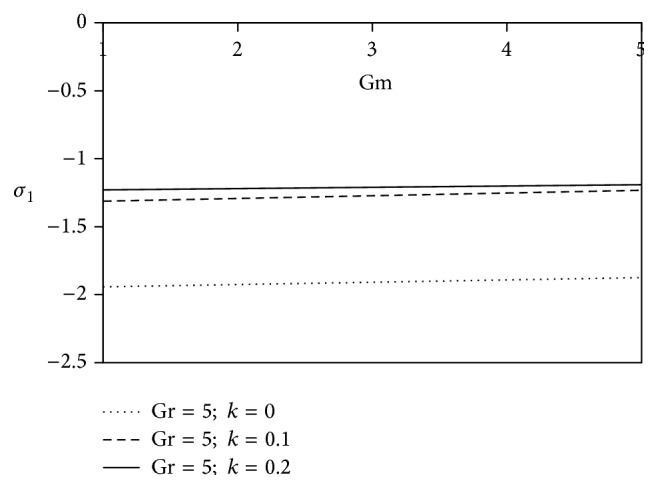
Variation of axial skin friction *σ*_1_ against Gm for Pr = 3, *M* = 2, Sc = 5, *γ* = 0.5, Be = 1, and Bi = 1.

**Figure 19 fig19:**
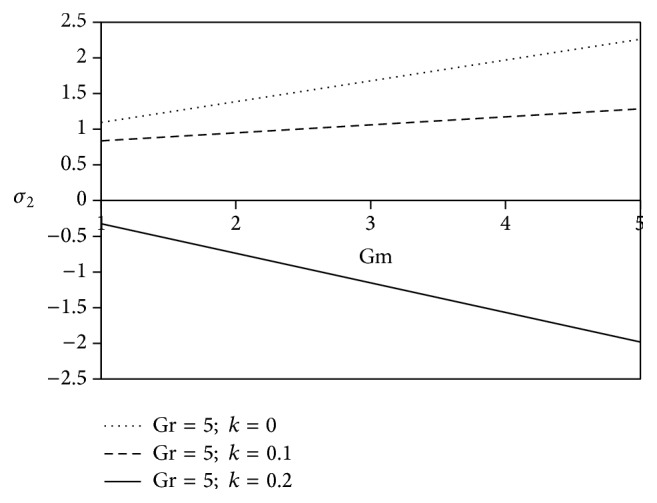
Variation of transverse skin friction *σ*_2_ against Gm for Pr = 3, *M* = 2, Sc = 5, *γ* = 0.5, Be = 1, and Bi = 1.

**Figure 20 fig20:**
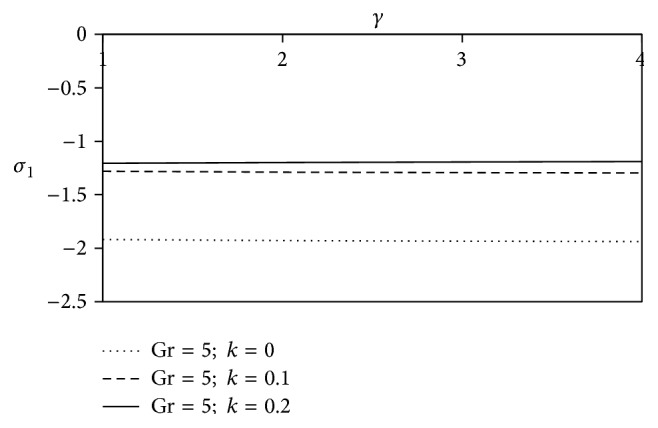
Variation of axial skin friction *σ*_1_ against *γ* for Pr = 3, *M* = 2, Sc = 5, Gm = 5, Be = 1, and Bi = 1.

**Figure 21:**
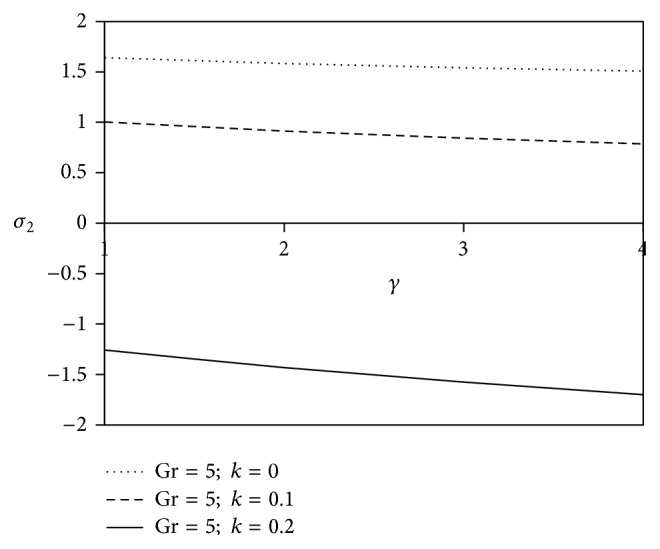
Variation of transverse skin friction *σ*_2_ against *γ* for Pr = 3, *M* = 2, Sc = 5, Gm = 5, Be = 1, and Bi = 1.

The objective of the present paper is to investigate the effects of ion slip on viscoelastic fluid flow past an impulsively started infinite vertical plate embedded in a porous medium with chemical reaction. In order to get a physical insight of the effects of flow parameters on the flow problem under consideration, we make graphical illustration for velocity field in Figures 2 [Fig fig3] [Fig fig4] [Fig fig5] [Fig fig6] [Fig fig7] [Fig fig8] [Fig fig9] [Fig fig10]–11 and shearing stress in Figures 12 [Fig fig13] [Fig fig14] [Fig fig15] [Fig fig16] [Fig fig17] [Fig fig18] [Fig fig19] [Fig fig20]–21. The parameters *K* = 1, *ωt* = *π*/2 are kept fixed throughout the discussion. The nonzero values of the parameter *k* characterize the viscoelastic fluid and *k* = 0 represents the Newtonian fluid flow phenomenon.

Figures 2 and 3 reveal the effects of both the magnetic parameter *M* and the viscoelastic parameter *k* on the fluid velocity components *u* and *w*. In general, application of transverse magnetic field has the tendency to decrease the velocity due to resistive Lorentz force. In both of the figures, the enhancement of viscoelasticity modifies the primary fluid velocity *u* and secondary fluid velocity *w* in comparison with Newtonian fluid flow phenomenon. Also, the growth of magnetic parameter *M* shifts the fluid velocity in downward direction for both of the velocity components.

Grashof number studies the behavior of free convection and it is defined as the ratio of buoyancy force to viscous force. It plays an important role in heat and mass transfer technology. In this study, the results are discussed for the flow past an externally cooled plate (Gr > 0) and flow past an externally heated plate (Gr < 0). Figures 4 and 5 are depicted for positive values of the buoyancy parameter Gr which corresponds to the cooling problem. The cooling problem is often encountered in the cooling of nuclear reactors. It is experienced from Figures 4 and 5 that the rise of Grashof number (Gr) with the increasing values of viscoelastic parameter magnifies fluid flow velocity in both types of velocity components.

Figures 6 and 7 exhibit the effects of viscoelastic parameter on fluid velocity for the flow past a heated plate (Gr < 0) and for the flow past a cooled plate (Gr > 0). It is seen from the figures that, for the flow past a heated plate, the flow nature of Newtonian fluid diminishes as compared to the flow past a cooled plate. Furthermore, with the amplification of viscoelastic parameter, the velocity pattern for heated plate gradually decreases in comparison with Newtonian fluid but a reverse trend is observed in case of cooled plate for both primary and secondary velocity components.

Figures 8 and 9 represent the graphs of primary and secondary velocity components for different values of viscoelastic parameter (*k*) when the effect of Hall current is accounted. From these figures, we note that the enhancement of Hall current declines the trend of both primary and secondary velocity profiles along with the increment of viscoelastic parameter.

Figures 10 and 11 depict the influence of ion slip parameter Bi on fluid velocity with viscoelastic parameter. Analyses of the graphs show that the rising effect of ion slip parameter is to increase the velocity profiles for both Newtonian and non-Newtonian cases.

After knowing the velocity field, it is very important from a physical point of view to know the effect of viscoelastic parameter on resistive force or viscous drag. The resistive force or viscous drag on the surface of the body due to the motion of the fluid is known as the shearing stress. To get the physical behavior of the axial shearing stress and transverse shearing stress, Figures 12 [Fig fig13] [Fig fig14] [Fig fig15] [Fig fig16] [Fig fig17] [Fig fig18] [Fig fig19] [Fig fig20]–21 have been plotted to notify the nature of shearing stress formed at the externally cooled surface (Gr > 0) with the variation of viscoelastic parameter (*k*) for different values of flow parameters: Prandtl number (Pr), Hall parameter (Be), ion slip parameter (Bi), radiation (R), Grashof number for mass transfer (Gm), and chemical reaction parameter (*γ*).

Prandtl number plays a significant role in heat transfer flow problems as it helps to study the simultaneous effects of momentum and thermal diffusion in fluid flow. Figures 12 and 13 represent the nature of axial and transverse components of shearing stress experienced by Newtonian as well as non-Newtonian fluid against Prandtl number. Analysis of the graph shows that the magnitude of the axial shearing stress rises with the variation of viscoelastic parameter, but, on the other hand, a reverse trend is observed in case of transverse shearing stress.

Figures 14 and 15 have been plotted to discuss the axial and transverse shearing stress against Hall parameter (Be). It is seen that near the surface of the plate, the magnitude of axial shearing stress for non-Newtonian fluid gradually increases as compared to Newtonian fluid, but a descending pattern is encountered in case of transverse shearing stress.

The effects of ion slip parameter (Bi) on the shearing stress for Newtonian as well as non-Newtonian fluids at the plates are revealed in Figures 16 and 17. From these graphs, we observe that the rising trend of ion slip parameter shows a growth in axial shearing stress along with the increasing values of *k* in comparison with Newtonian fluid, but a reverse physical nature is observed for transverse shearing stress.

The consequences of free convection parameter for mass transfer (Gm) are noticed in Figures 18 and 19. Gm > 0 indicates that the free stream concentration is less than the concentration at the boundary surface. These figures mainly discuss the nature of resistive force of fluid flow during the various positive values of Grashof number for mass transfer (Gm). The figures reveal that the increasing values of Gm lead to subdue the magnitude of axial shearing stress for Newtonian fluid in comparison with viscoelastic fluid. Furthermore, the transverse shearing stress for Newtonian fluid accelerates whereas resistivity diminishes away from the plate for viscoelastic fluid.

Figures 20 and 21 display the variation of shearing stresses against chemical reaction parameter *γ* with viscoelastic parameter. The graphs enable the fact that as the chemical reaction parameter *γ* increases, the resistivity of the non-Newtonian fluids is enriched for axial shearing stress and there would be a significant decrease of magnitude in case of transverse shearing stress away from the surface as compared to simple fluid.

## 4. Conclusion

The effects of ion slip on unsteady MHD flow of an incompressible electrically conducting viscoelastic fluid past an impulsively started vertical plate embedded in a porous medium with chemical reaction in presence of heat and mass transfer are studied in this paper. Some of the important conclusions of this paper are as follows.

- The flow field is significantly affected with the variation of viscoelastic parameter.
- The effect of ion slip parameter on velocity is prominent throughout the boundary layer in presence of other flow parameters.
- The rates of heat transfer, that is, Nusselt number, and rate of mass transfer, that is, Sherwood number, are not significantly affected during the variations of viscoelastic parameter throughout the fluid flow phenomenon.
- The axial and transverse components of shearing stress are prominently affected by the viscoelastic parameter along with other flow parameters.

## Nomenclature

(*x*′, *y*′, *z*′): Cartesians coordinates
(*x*, *y*, *z*): Dimensionless Cartesian coordinates
(*u*′, *v*′, *w*′): Velocity components along the (*x*′, *y*′, *z*′) directions, respectively (m s^−1^)
*U*_0_: Plate velocity (m s^−1^)
*V*_0_: Suction velocity (m s^−1^)
*u*: Dimensionless primary velocity
*w*: Dimensionless secondary velocity
*T*′: Temperature (K)
*T*_*∞*_′: Ambient temperature (K)
*ϕ*′: Species concentration at any point in the fluid (kg m^−3^)
*ϕ*_*∞*_′: Free stream concentration
*D*: Mass diffusion coefficient (m^2^ s^−1^)
*ϕ*: Dimensionless concentration
*t*′: Time
*t*: Dimensionless time
*ω*′: Frequency of oscillation
*ω*: Dimensionless frequency of oscillation
*K*: Porous medium permeability
*η*_0_: Dynamic viscosity (kg m^−1^ s^−1^)
*k*: Viscoelastic parameter
*θ*: Dimensionless fluid temperature
*ν*: Kinematic viscosity (m^2^ s^−1^)
*K*_*l*_: Dimensionless chemical reaction parameter
*γ*: Chemical reaction parameter
*ρ*: Density (Kg m^−3^)
*β*: Volumetric coefficient of thermal expansion (K^−1^)
*a*, *b*: Constants
Bi: Ion slip parameter
Be: Hall parameter
*β*^*^: Volumetric coefficient of mass expansion (K^−1^)
*K*_*T*_: Thermal conductivity of the fluid (W K^−1^ m^−1^)
*C*_*p*_: Specific heat at constant temperature (J Kg^−1^ K^−1^)
*σ*_1_: Dimensionless axial shear stress
*σ*_2_: Dimensionless transverse shear stress
*σ*: Electrical conductivity ((ohmm)^−1^)
*g*: Acceleration due to gravity (m s^−2^)
*B*_0_: External magnetic field
*θ*_0_: Dimensionless zeroth-order temperature profile
*ϕ*_0_: Dimensionless zeroth-order concentration profile
*M*: Magnetic parameter
Gr: Thermal Grashof number
Pr: Prandtl number
Sc: Schmidt number
Gm: Concentration Grashof number
Nu: Nusselt number
Sh: Sherwood number.
